# Editorial: Case reports in heart failure and transplantation: 2023

**DOI:** 10.3389/fcvm.2024.1359393

**Published:** 2024-01-23

**Authors:** Matteo Cameli, Federico Landra

**Affiliations:** Department of Medical Biotechnologies, Division of Cardiology, University of Siena, Siena, Italy

**Keywords:** acute myocadial infarction, mechanical complication of myocardial infarction, mechanical circulatory support, congenital heart defect, pericarditis

**Editorial on the Research Topic** Case reports in heart failure and transplantation: 2023

In this editorial we summarize some of the contributing articles to the Research Topic “Case Reports in Heart Failure and Transplantation: 2023” of the journal Frontiers in Cardiovascular Medicine.

## Old but gold

Nowadays incidence of ventricular septal rupture following acute myocardial infarction (*post-AMI VSR*) has considerably decreased thanks to the capillary diffusion of CathLabs and effective treatment strategies, with less than 1 out of 300 patients suffering from AMI developing it. However, despite adequate management, post-AMI VSR remains a serious complication with an extremely high risk of mortality, as high as 40%–50%, a number that has not decreased over time (doi:10.1001/jamanetworkopen.2021.28309). Almost half of patients with a post-AMI VSR presents with cardiogenic shock.

Management of post-AMI VSR is anything but easy. Despite being surgery the treatment of choice for mechanical complications of AMI such as VSR, there is now increasing recognition of the potential benefit of employing mechanical circulatory support systems (*MCS*) to stabilize hemodynamics and postpone as much as possible the surgical repair. Indeed, better results from surgical repair have been demonstrated for patients who have undergone surgery at least 7 days after the occurrence of the VSR, as the myocardial tissue surrounding the VSR should have the time to mature and heal. Therefore, even the latest ‘*2023 ESC Guidelines for the management of acute coronary syndromes*’ suggest this strategy, with an increasing indication to MCS in the very early stages.

Of note, intra-aortic balloon pump (*IABP*) is not considered an MCS at all, nonetheless is extremely helpful in reducing left ventricular afterload and, therefore, the amount of left-to-right shunt. Indeed, IABP has a IIa recommendation in patients with AMI-related mechanical complications, including papillary muscle rupture. When IABP alone or combined with drugs such as vasodilators, inotropes and diuretics is not enough to reach a stable hemodynamic condition, more aggressive MCS can be delivered.

Guidelines do not recommend a specific type of MCS to be preferably employed. Each one has its own advantages and disadvantages. For instance, *Impella* has a relative contraindication for its use in the setting of post-AMI VSR because of the risk of tissue ingestion and the possible shunt reversal (right-to-left), but surely is more physiological than ECMO. On the contrary, *ECMO* may be a valuable solution but it must be considered that it completely overturns the normal cardiovascular physiology. Particularly, ECMO increases left ventricular afterload and reduces right sided pressures, therefore significantly increasing the left-to-right shunt and oxygen demand-supply ratio. A recent study by Burkhoff and collogues evaluated the possible impact of the different MCS to hemodynamics in the setting of VSR through a computational simulation model. They concluded that Impella provides the best hemodynamic improvement in post-AMI VSR, reducing pulmonary capillary wedge pressure and shunt (doi: 10.1161/CIRCHEARTFAILURE.119.005981).

However, aside from the MCS chosen to support hemodynamics, even when the patient gets to surgery, it does not mean that all the problems are gone. On the contrary, mortality still remains high and there is always the possibility of VSR recurrence/enlargement, as in the case reported by Garg et al. in the present Research Topic.

In this case, recurrent VSR was successfully treated percutaneously, sparing the need for another surgical intervention. Nonetheless, the patient did not recover from right heart failure. Therefore, she was discussed by heart failure specialists and final decision on listing her for heart transplantation was made. Of note, guidelines recommend heart transplant in cases in which VSR recurs and re-operation is unlikely to be successful, but the Authors showed that percutaneous VSR repair is still feasible at this stage. However, heart transplant probably remained the only available option here since, despite adequate treatment of post-AMI VSR, refractory right heart failure persisted.

## Until surgeon do us apart

Remaining in the context of clinical conditions which lead to volume overload of the right sided chambers, Bou Chaaya et al. presented an interesting case of a patient with concomitant partial anomalous pulmonary venous return (*PAPVR*) and constrictive pericarditis. The diagnosis of constrictive pericarditis here was not straightforward, since the right sided chambers were enlarged and cardiac magnetic resonance did not show a thickened pericardium. However, it should be remembered that up to 1 out of 5 patients can have a normal pericardium despite having a constrictive pericarditis (doi: 10.1161/01.CIR.0000087606.18453.FD). Also, the presence of septal bounce on echocardiography, the equal right ventricular and left ventricular diastolic pressures and the ‘*dip and plateau’* pattern at cardiac catheterization were suggestive.

Of note, cardiac catheterization is recommended by ‘*2015 ESC Guidelines for the diagnosis and management of pericardial diseases*’ in cases in which non-invasive examinations provide conflicting results and not in all cases. In this case cardiac catheterization was performed appropriately, and was also helpful to obtain an angiogram of the PAPVR. Surgery confirmed both diagnoses.

PAPVR is a rare congenital heart anomaly that may remain unnoticed for a long time. In this case it was responsible for a *Qp:Qs* of 1,35:1, which is actually under the recommended cut-off for surgical intervention. However, the patient was symptomatic and a concomitant constrictive pericarditis were good points for surgical intervention. Therefore, the patient underwent surgery with subtotal pericardiectomy and concomitant Warden procedure due to the high implantation of the anomalous right upper pulmonary vein return. Interestingly, the pericardium during the surgical intervention was reported being thickened and constrictive, differently from cardiac magnetic resonance description. Subsequent histological examination actually revealed the presence of fibrosis and calcification.

The Authors comment that probably the two conditions, PAPVR and constrictive pericarditis, do not have a cause-effect relationship, since this bizarre association has been only reported once before them. Therefore, they lived together in this unique case only “until the surgeon did them apart”.

## Final considerations

This Research topic included some very interesting case reports which highlighted either unique solutions to common problems or common solutions to very rare conditions. Each case report is potentially useful to others to solve a brain teaser with more confidence and safety. Therefore, readers are strongly encouraged to further submit their most interesting clinical cases to reach this noble aim. Aside from the cases highlighted herein, two other high-quality cases have been published in this Research Topic which well deserve a lecture from each heart failure enthusiast.

